# Associations between sporting physical activity and cognition in mid and later‐life: Evidence from two cohorts

**DOI:** 10.1111/sms.14412

**Published:** 2023-05-30

**Authors:** J. J. Mitchell, M. Hamer, J. M. Blodgett, G. S. Wannamethee, B. J. Jefferis

**Affiliations:** ^1^ Department of Primary Care and Population Health Upper Third Floor UCL Medical School (Royal Free Campus) London UK; ^2^ Division of Surgery and Interventional Sciences, Faculty Medical Sciences, Institute of Sport Exercise & Health University College London London UK

**Keywords:** accelerometer, cognition, executive function, exercise, leisure activity, memory, physical activity, sporting

## Abstract

Evidence has linked sporting leisure time physical activity (sporting‐LTPA) to healthy cognition throughout adulthood. This may be due to the physiological effects of physical activity (PA), or to other, psychosocial facets of sport. We examined associations between sporting‐LTPA and cognition while adjusting for device‐measured PA volume devoid of context, both in midlife (*N* = 4041) participants from the 1970 British Cohort Study and later‐life (*N* = 957) participants from the British Regional Heart Study. Independent of device‐measured PA, we identified positive associations between sporting‐LTPA and cognition. Sports with team/partner elements were strongly positively associated with cognition, suggesting LTPA context may be critical to this relationship.

## 1 INTRODUCTION

Leisure time activity engagement, defined as activities involving mentally stimulating, social or physical components,^1^ is increasingly recognized as protective against cognitive decline.^1^, ^2^, ^3^, ^4^, ^5^ Physical activity (PA) when assessed through questionnaires or objectively with accelerometers is associated with healthy cognition^6^ and healthy cognitive aging.^7^ The 2019 Copenhagen Consensus Statement on PA and aging highlights moderate intensity PA as being favorable, but concedes a lack of evidence exists as to the importance of PA context in relation to cognitive reserve.^8^ Leisure time PA (LTPA) can vary greatly in the degree of overlap between social, physical, and mental domains. Distinguishing the other psychosocial benefits of LTPA, and specifically sporting, partner or team‐focused LTPA plays from the purely physiological effects of increased movement is a critical difference which could inform the optimal dose and type of PA recommendations for healthy cognition.

Current evidence which assesses associations of PA of different forms with cognition are limited, focusing principally on later‐life samples,^1^ when prodromal cognitive decline or dementia onset is abundant, and does not distinguish between bodily movement and the other psychosocial domains of leisure activity.^1^, ^2^, ^5^

This study explores whether sporting‐LTPA is associated with cognition and whether this is independent from device‐measured PA volume. A key aspect is to explore whether these relationships differ for partner/team‐based activities. We conducted our analyses in two distinct cohorts of old age and midlife adults to address the issue of prodromal cognitive decline.

## 2 METHODS

Midlife participant data were drawn from the age‐46 follow‐up of the 1970 British Cohort Study (BCS70), a birth cohort all born within a single week.^9^ Later‐life participant data were drawn from the 30‐year follow‐up of the British Regional Heart Study (BRHS) and consisted of community‐dwelling men aged 71–91 at the study's 30‐year follow‐up.^10^ Participants undertook self‐completion questionnaires, computer‐assisted interviewing, and nurse biomedical assessments. All participants gave written informed consent and the BCS70 study received ethical approval from the National Research Ethics Service (NRES) Committee South East Coast—Brighton and Sussex (Ref 15/LO/1446), and BRHS from the NRES Committee for London (MREC/02/02/91). Inclusion criteria were limited to participants who provided all relevant measures.

### 2.1 Cognition outcomes

In BCS70, an abbreviated subset of memory and executive function tests were measured using computer‐administered tests involving: immediate and delayed recall of a 10‐word list; a verbal fluency task involving naming as many animals as possible in a 1‐minute interval; and a letter‐cancellation task in which participants screen a grid of letters and eliminate any “*P's”* and “*W's”*.^5^, ^11^, ^12^ The number of letters screened is a measure of speed and the count of letters missed is a score of accuracy. All five scores were converted into z‐scores and summed.

In BRHS, a validated tool of global cognition (Test Your Memory; TYM)^13^ was used to measure cognition and similarly tests both memory and executive function. Test scores were also converted into z‐scores.

### 2.2 Sporting‐LTPA

In BCS70, sporting‐LTPA was derived from a subset of the EPIC‐Norfolk PA questionnaire.^14^ Participants were asked whether they participated in a number of sporting activities such as “*swimming, mountaineering, cycling, aerobics, weight training, dance, running etc”* with possible responses “*none”*, “*less than monthly”, “monthly”, “2–3 times/month”, “weekly”, “2–3 times/week”, and “6+ times/week”* for each sport (see Table S1). Using the midpoints of each activity as a count of session frequency, new categories were derived from the summed frequency across all sports, and encompassed, “*none”*, “*<monthly”, “1–4 times/month”, “2‐4 times/week”, “5+ times/week”*.

BRHS participants were asked “*How many times per month do you take active sporting physical exercise such as running, swimming, dancing, golf etc.?”* with possible answers “None”, “Occasionally (less than monthly)”, and “Frequently (once a month or more)” followed by reporting the session frequency. Frequency responses were categorized in alignment with BCS70.

### 2.3 Covariates

Covariates were chosen based on previous literature (Table S1) and included sex, age, region, education, a socioeconomic indicator, disability, other social engagement, psychological distress, smoker status, alcohol consumption, and BMI. Moderate and vigorous physical activity was measured using a hip‐worn Actigraph GT3X accelerometer device for waking time in BRHS (ActiGraph, Pensacola, FL), and a thigh‐worn activPAL3 device without removal in BCS70 (activPAL3 micro; PAL Technologies Ltd., Glasgow, UK; see Table S1).^15^

### 2.4 Statistical analysis

Given BRHS is comprised of male participants and analyses in BCS70 were stratified by sex. Within cohorts, multiple linear regression was used to assess the associations between sports participation and cognition z‐scores. Gradually mounting adjustments were made for potential confounders: (i) *age* (BRHS only), (ii) sociodemographic factors (iii) health and lifestyle factors, (iv) accelerometer‐derived PA and lastly, and (v) other social engagements.

### 2.5 Sensitivity analyses

Sports were recharacterized by their degree of social contact. To account for the heterogeneity in social interaction within sports themselves, only sports with overt partner or team elements (*mountaineering, golf*, *bowling*, *dancing*, *tennis*, *table‐tennis*, *rowing*, *squash*, *football*, *netball*, *snooker*, *wrestling*, *cricket*) were considered team/partner‐based (see Table S1).

Analyses in BRHS were repeated using the defined TYM cut point for cognitive impairment to align with the tool's validated protocol,^13^ utilizing a logistic regression approach. Further analyses re‐examined the fully adjusted models with total PA in place of MVPA.

## 3 RESULTS

Our midlife sample consisted of 4041 BCS70 participants (*N* = 2035 Male; *N* = 2006 Female) aged 46. Our later‐life sample included 957 male BRHS participants of median age 77 (IQR:74–81; Figures S1 and S2). Sporting‐LTPA frequency was highest in BCS70 with 76% of female participants and 81% of male participants engaging in sports at least monthly, compared to 34% of later‐life BRHS participants. In BCS70, cognition, specifically memory, was highest in females (Table S3). Further sample characteristics are presented in Tables S2–S4.

**FIGURE 1:**
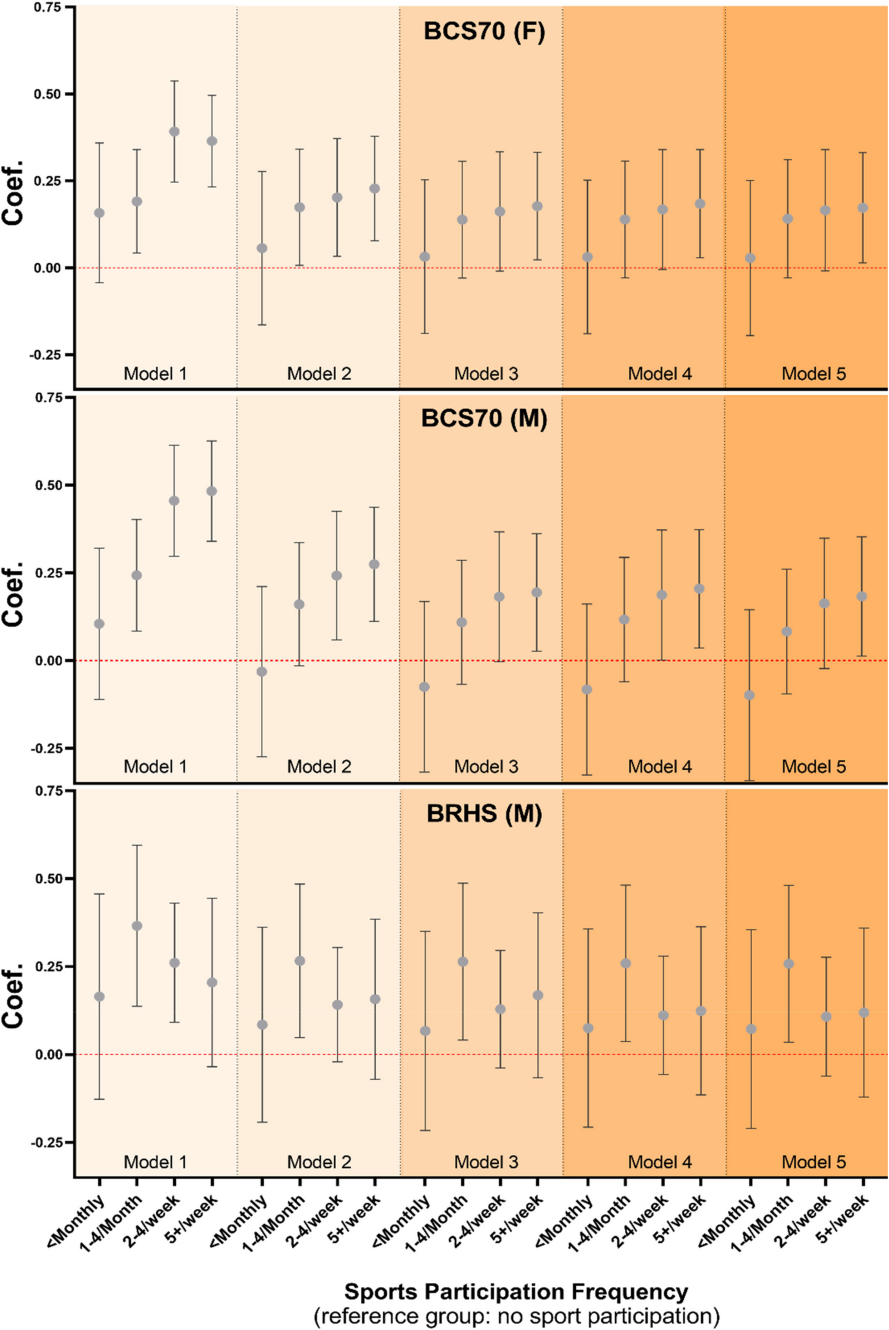
Associations of sporting‐LTPA and cognition z‐scores. Model 1 adjusts for age (excl. BCS70). Model 2 additionally adjusts for socioeconomic factors including socioeconomic class (BRHS: social class), region, and education. Model 3 additionally adjusts for health factors including disability status, BMI, history of stroke, CVD, hypertension, diabetes, smoker status, and alcohol consumption. Model 4 additionally adjusts for physical activity, wear time, and wear season. Model 5 additionally adjusts for other social engagement time.

Greater sporting‐LTPA was positively associated with cognition z‐scores, relative to no participation in both cohorts (Figure 1, Tables S5–S10). Adjusting for device‐measured PA made minimal change to the observed associations. In BCS70 male participants, sporting‐LTPA engagement 2–4 times/week (*β* = 0.19; 95%CI: 0.002–0.37; Figure 1) or 5+ times/week (*β* = 0.21; 95%CI: 0.03–0.37) was positively associated with cognition relative to no sporting‐LTPA after adjustment for device‐measured PA. Participating 5+ times/week remained positively associated with cognition after full‐adjustment in both BCS70 male (*β* = 0.18; 95%CI: 0.01–0.35) and female participants (*β* = 0.17; 95%CI: 0.01–0.33). In later‐life, participation 1–4 times/month proved associated with cognition, relative to no participation after full adjustment (*β* = 0.26; 95%CI: 0.04–0.48).

### 3.1 Sensitivity

**FIGURE 2:**
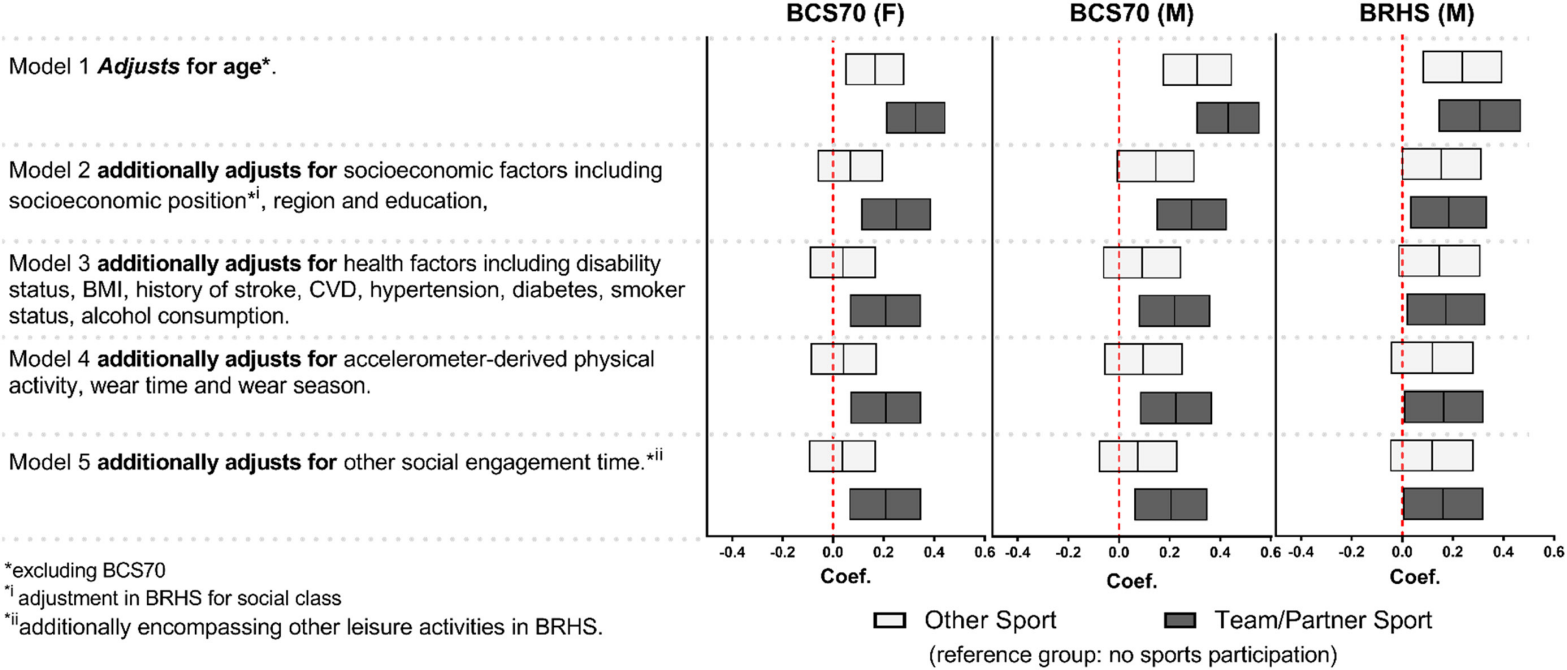
Association of team/partner sporting‐LTPA and cognition z‐scores.

Team/partner sporting‐LTPA proved more strongly associated with cognition than other types of sporting activities (Figure 2). After full adjustment, participation in team/partner sports remained positively associated with cognition relative to no sporting‐LTPA both in BCS70 males (*β* = 0.21; 95%CI: 0.06–0.35; Figure 2) and females (*β* = 0.21; 95%CI: 0.06–0.35), and in BRHS male participants (*β* = 0.16; 95%CI: 0.002–0.32).

When repeating analyses in BRHS participants using the validated TYM cut points revealed stronger patterns of association (Tables S11 and S12). Further, adjusting for total device‐measured PA, rather than MVPA only revealed similar patterns of association (Tables S13–S18, Figure S3).

## 4 DISCUSSION

We aimed to characterize the relationship between sporting‐LTPA and cognition in mid and later‐life. We report positive associations between context‐specific sporting‐LTPA and cognition after adjustment for context‐devoid device‐measured PA (both MVPA and total PA). These findings suggest that associations between sport and cognition may in part be driven by pathways which are independent from the physiological effects of exercise volume, such as psychosocial mechanisms. It has been observed that sporting activities may vary in their cognitive demands,^16^ which may lead to differences in any downstream cognitive benefits and may provide just one possible mechanism beyond the known physiological responses to MVPA.^17^, ^18^ In midlife, the relationship between sport participation and cognition appears strongest for greater volumes of sport (participating 5+ times/week). In older adults however, more modest volumes (1–4 times/month) appeared most strongly associated with cognition.

These findings align with previous studies which report sport as conferring improvements to cognitive performance, but is now substantiated with adjustments for device‐measured PA.^19^ Evidence is sparse as to the pathways by which sporting‐LTPA may impact cognition and does not pinpoint increased cardiorespiratory fitness as being the mediator of this relationship.^20^ Instead, emerging evidence now focusses on the anti‐inflammatory role of exercise as one possible pathway.^20^ We posit an additional role for sport in promoting cognitive‐stimulation and social engagement,^20^, ^21^, ^22^, ^23^ supported by our findings that team/partner sports appear most favorable. These associations did vary in robustness by age and sex; however, proving most robust in midlife male participants. This may be due to lower participation among female and later‐life participants in this sample and subtle differences in the types of activities being engaged in between ages and genders. Nonetheless, our findings may suggest that PA guidelines encouraging group‐based sporting activities may yield additional benefits beyond targeting increased movement.

### 4.1 Strengths/Limitations

This study utilized two large samples at different life‐stages, closely harmonized to explore the study aims. Use of device‐measured PA also effectively distinguished the physiological benefit of exercise from other aspects of sport. Nonetheless, both cohorts under‐represent non‐white communities, and BRHS is a solely male cohort, limiting the generalizability of these findings. Minor differences do also exist in the measures used between cohorts and how they were necessarily coded for brevity, including use of a tool of global cognition in later‐life, but an abbreviated subset of cognitive measures in midlife. Further, given the study's cross‐sectional nature, it is likely that this observed relationship is bidirectional, given in later life, cognitive impairment often follows physical frailty,^24^ which may precipitate lessened participation. This risk may be partly mitigated by our replication of findings in the midlife BCS70 cohort. Replication of these findings with repeated measures of cognition would provide greater insight into the value of sporting LTPA for slowing cognitive decline.

## 5 CONCLUSION

We identified positive associations between sporting‐LTPA and cognition both in mid and later‐life. Participation in team/partner sports was most strongly associated with cognition in midlife participants suggesting that sporting context may be important in shaping the associations.

## AUTHOR CONTRIBUTIONS

BJJ, MH, JMB, and JJM conceived the study. JJM and BJJ conducted analysis and interpretation. JJM drafted the manuscript. BJJ, GW, JMB, and MH examined final analyses and revised several drafts before all authors read and approved the final manuscript.

## FUNDING INFORMATION

This study was funded by a British Heart Foundation grant (SP/15/6/31397). JJM is funded by MRC grant (MR/N013867/1). JMB is supported through a British Heart Foundation grant SP/F/20/150002.

## Supporting information


Appendix S1

## Data Availability

Original study protocol and survey documents can be found online at: https://bcs70.info/ and access to this data is available through the UK data service: UK Data Service › Series. The datasets supporting this article are available in the UK Data Service repository [1970 British Cohort Study: https://beta.ukdataservice.ac.uk/datacatalogue/series/series?id=200001].
