# Supplementary material for: Associations between sporting physical activity and cognition in mid and later‐life: Evidence from two cohorts
Source: Scand J Med Sci Sports. 2023 May 30;33(8):1570–5. doi: 10.1111/sms.14412 (PMC10947539; doi:10.1111/sms.14412)
Supplement: Supplementary file 1 — Appendix S1 [file SMS-33-1570-s001.docx]

**Supplementary Material**

**Supplementary Figure S1. BCS70 Strobe Diagram**

**
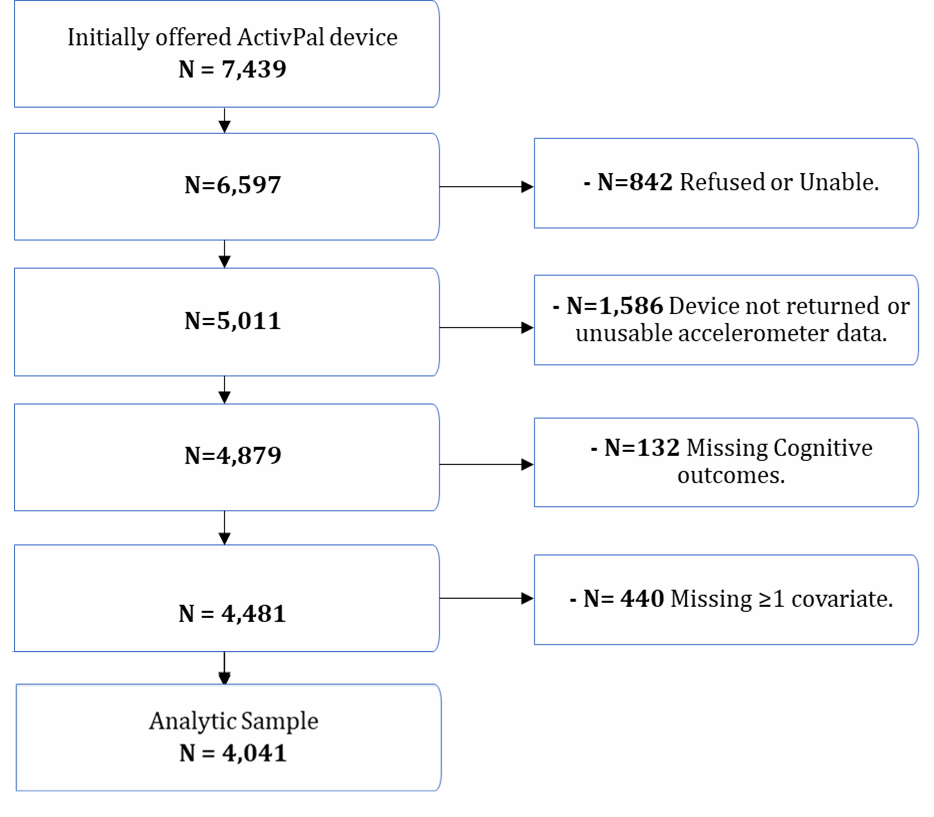
**

**Supplementary Figure S2. BRHS Strobe Diagram**

**
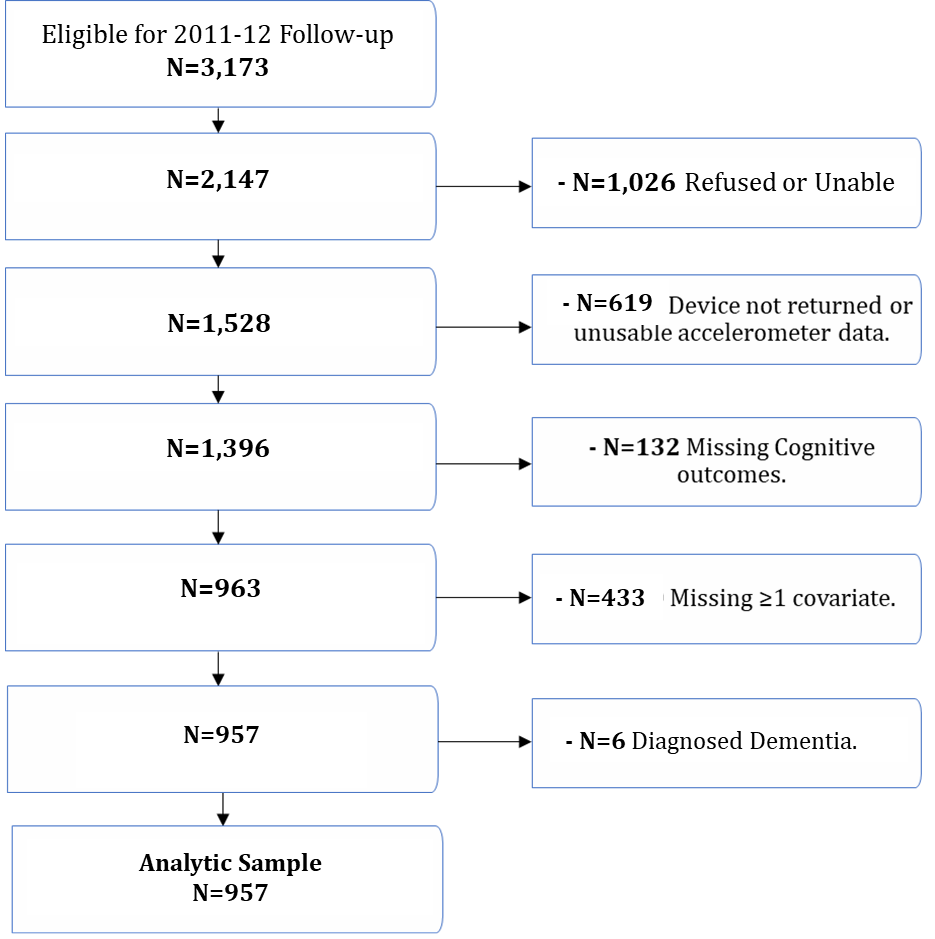
**

**Supplementary Table S1. Covariate coding by cohort.**

| **Covariate** | **BRHS** | **BCS70** |
| --- | --- | --- |
| Age | N/A |  |
| Region | Coded as Categorical: North of England, midlands, south of England and Scotland | Coded as Categorical: South (London, Channel Islands, South East, South West); Midlands (East Midlands, West Midlands, East of England); North (North West, North East, Yorkshire and the Humber, Isle of Man); Scotland; Wales; Northern Ireland. |
| Education | Categorised as <15 years, 15-17 years, and ≥18 years of age | Categorized as <15 years, 15-17 years, and ≥18 years of age |
| Socioeconomic Class | N/A | Coded as categorical, derived from NS-SEC and collapsed to: Routine/Semi-routine employment; Small employer/lower supervisory; Lower managerial/intermediate; High professional/managerial |
| Social Class | Coded as categorical based on participants’ longest held occupation, reported at baseline, and was categorised into non-manual (classes I, II and III non-manual) and manual (classes III manual, IV and V groups). | N/A |
| Smoking | Coded as categorical: never; ex-smoker (≥20y); ex-smoker (<20y); current smoker | Coded as categorical: Never; Ex-smoker; Current irregular smoker; Current regular |
| Alcohol | Self-reported. Coded as categorical: None; Non-risky (<14 weekly units); Risky (≥14 weekly units) | Self-reported using AUDIT-C scale coded as categorical: None; Non-risky (<14 weekly units); Risky (≥14 weekly units) |
| BMI | Derived from nurse measured weight and height. Coded as categorical: (normal or underweight BMI [<25.0]; BMI overweight [≥25.0 - 29.9]; BMI obese [≥30]) | Derived from nurse measured weight and height. Coded as categorical: (normal or underweight BMI [<25.0]; BMI overweight [≥25.0 - 29.9]; BMI obese [≥30]) |
| Stroke | Self-reported coded as binary | Self-reported coded as binary |
| CVD | Self-reported coded as binary No; Yes (MHx of ‘Acute coronary syndrome’, ‘Aortic Aneurysm’, ‘Heart attack’, ‘Heart Failure’, ‘Angioplasty’ or ‘Coronary artery bypass, ‘Other problems of the heart and circulation’.) | Self-reported coded as binary: No; Yes (Heart attack; Coronary heart disease, congestive heart failure) |
| Diabetes | Self-reported coded as binary. | Self-reported coded as binary |
| HTN/ Antihypertensive medication use | Self-reported coded as binary: No; Yes (Self-reported HTN or taking minimum 1 HTN medication) | Self-reported coded as binary: No; Yes (Self-reported HTN or taking minimum 1 HTN medication) |
| Limiting Disability | Presence of a limiting disability, measured using a 6-question scale encompassing whether participants report having difficulty, ‘going up or down stairs’, ‘going out of the house’, ‘keeping balance’, ‘bending down’, ‘straightening up’, and ‘walking 400 yards, on your own due to the long-term health problem’. Reporting difficulty with one or more of these tasks was scored as 1 on a binary yes/no variable. | Derived using the EU-SILC measure of disability which categorises participants as ‘none’, ‘somewhat limited’ or ‘severely hampered’ in their daily activities. This was collapsed to a binary variable of ‘any limitation’ and ‘none’. |
| Moderate and vigorous PA | Measured using the Actigraph GT3X (ActiGraph, Pensacola, FL) worn on participants hips for a maximum of 7 days and a minimum of 3 full days (600 complete minutes of wear time across 3 days in the data collection window) removed only for bathing or swimming. Actigraph counts per minute (CPM) was recoded into mean daily minutes spent in light intensity PA, moderate to vigorous PA and SB. Episodes of extreme high CPM (>10,000) or step count (>20,000) were corroborated against the participant activity log. Episodes of sustained SB (>90 minutes of 0 CPM) and only minimal movement artifact were considered as non-wear time [60]. The cut-point for LIPA was considered 100-1040 CPM, ≥1040 CPM for MVPA and <100 CPM for SB. Mean time in, MVPA was calculated from overall daily wear time across the three-day period [61] and included as hours. | PA, SB and sleep time were measured using a thigh-mounted accelerometer device worn continuously for up to seven days including for sleeping and bathing in line with the validated wear protocol (activPAL3 micro; PAL Technologies Ltd., Glasgow, UK). MVPA time was derived using a step cadence of ≥10055. Light intensity PA (LIPA) was derived as the residual from total movement activity. SB was defined as non-sleep time spent sitting or lying. MVPA was included in hours. |
| Sport participation | Self-reported, coded as categorical. ‘Do you take active sporting physical exercise such as running, swimming, dancing, golf, tennis, squash, jogging, bowls, cycling, hiking etc.?’ with possible answers, ‘No’, ‘Occasionally (less than monthly)’ and ‘Frequently (once a month or more)’ and 'How many hours per month'. In alignment with BCS70, Monthly prevalence was then operationalised as ‘none (0 sessions)’, ‘less than monthly (0.5 sessions)’, ‘1-4 times/month (1-4 sessions)’, ‘2-3 times/week(5-12 sessions)’ and ‘4+ times/week (12 or more sessions)’ | Self-reported, coded as categorical; EPIC-Norfolk PA questionnaire. Participants were asked whether they participated in ‘swimming, , mountaineering, cycling, aerobics, weight training, conditioning, yoga, dance, running, jogging, bowling, tennis, squash, table tennis, golf, football, cricket, rowing, netball, fishing, horse-riding, snooker, ice-skating, sailing or wrestling’ with possible responses ‘none’, ‘less than monthly’, ‘monthly’, ‘2-3 times/month’, ‘weekly’, ‘2-3 times/week’ '4-5 times/week', ‘6+ times/week’. Mid-points of each category were used to calculate a monthly frequency of participation, and ‘less than monthly’ was recategorized as 0.5 times/month.  Monthly prevalence was then operationalised as ‘none (0 sessions)’, ‘less than monthly (0.5 sessions)’, ‘1-4 times/month (1-4 sessions)’, ‘2-3 times/week(5-12 sessions)’ and ‘4+ times/week (12 or more sessions)’ |
| Social Participation | Self-reported, coded as numeric: Participants were asked whether or not they spent time participating in social activities such as, ‘looking after grandchildren’, ‘spending time with family, friends and neighbours’, ‘talking with friends/relatives on the telephone’, ‘in paid work’, ‘in voluntary work’, ‘in a pub or club’, ‘attending religious services’, ‘playing cards, games, or bingo’, ‘visiting the cinema/restaurants/sporting events’ or ‘attending a class or course of study’. Participant’s responses were dichotomised into participation in at least 1-weekly hour versus none, and for sensitivity analyses were re-categorised into 0 weekly hours, 1-6 weekly hours, 7-13 weekly hours, 14-20 weekly hours and >21 weekly hours, reflecting 3 hours daily or more. | Self-reported, coded as categorical: Participants were asked 'How often do you see:' 'your family' and 'your friends'. With responses ‘Three times a week or more’, 'Once or twice a week'; Once or twice a month; Every few months; Once or twice a year; Less than once a year'; 'Never'; For family members an additional category was: 'I live with all family members'. For frequency of meeting friends, an additional category was 'I do not have any friends'.  Due to small numbers in the extreme categories for Family, ‘Never’ was combined with ‘Less than once a year’ and ‘Lives with family’ was grouped with ‘Three times a week or more’ for brevity. |

| **Supplementary Table S2** |  |  |  |  |
| --- | --- | --- | --- | --- |
| **Participant demographics grouped by Test Your Memory Score.** | | | |  |
|  |  | **BCS70 (Female)** | **BCS70 (Male)** | **BRHS (Male)** |
| **Sociodemographic Characteristics** |  | ***N=2006*** | ***N=2,035*** | ***N=963*** |
| **Age** | **Median (IQR)** | 46 (-) | 46 (-) | 77 (74-81) |
| **Region** | **%** |  |  |  |
| South |  | 31% (626) | 29% (592) | 36% (342) |
| Midlands |  | 25 (511) | 29% (594) | 16% (153) |
| North |  | 29% (584) | 28% (559) | 39% (373) |
| Scotland |  | 9% (182) | 9% (180) | 9% (89) |
| Wales |  | 5% (103) | 5% (110) | - |
|  |  |  |  |  |
| **Age Left Education** | **%** |  |  |  |
| <15 years |  | 54% (1082) | 59% (1211) | 21% (201) |
| 15 to 17 years |  | 6% (127) | 6% (109) | 58% (559) |
| >18 years |  | 40% (797) | 35% (715) | 21% (197) |
| **Social Class (Upper/Non-manual)** | **%** | - | - | 55% (528) |
| **Socioeconomic Class** |  |  |  |  |
| Routine/Semi-routine employment |  | 18% (363) | 15% (296) | - |
| Small employer/lower supervisory |  | 11% (222) | 24% (493) | - |
| Lower managerial/intermediate |  | 57% (1148) | 37% (747) | - |
| High professional/managerial |  | 14% (273) | 24% (499) | - |
| **Health Status and Behaviours** |  |  |  |  |
| **Smoker status** | **%** |  |  |  |
| Never smoke |  | 51% (1026) | 50% (1022) | 39% (375) |
| Ex-smoker |  | 32% (649) | 32% (655) | 57% (479) |
| Current Smoker |  | 17% (331) | 18% (358) | 3% (33) |
|  |  |  |  |  |
| **Alcohol Consumption** | **%** |  |  |  |
| None |  | 10% (201) | 7% (143) | 28% (272) |
| Occasional |  | 75% (1496) | 62% (1265) | 30% (287) |
| Daily/Most days |  | 15% (309) | 31% (627) | 42% (398) |
| **BMI** | **%** |  |  |  |
| Normal or Underweight |  | 37% (749) | 22% (438) | 30% (289) |
| Overweight |  | 32% (643) | 44% (905) | 52% (495) |
| Obese |  | 31% (614) | 34% (692) | 18% (173) |
| **Physical Activity Intensity (average daily mins over wear week)** | **Mean, SD** |  |  |  |
| Moderate or Vigorous Physical Activity |  | 52 (24) | 51 (26) | 41 (32) |
| Average Daily Actigraph Wear Time (Mins) |  | 942 (76) | 960 (75) | 855 (65) |
| **Sport Participation** | **%** |  |  |  |
| None |  | 17% (323) | 13% (247) | 61% (579) |
| <monthly |  | 7% (129) | 6% (121) | 5% (48) |
| 1-4 times/month |  | 19% (354) | 20% (379) | 9% (82) |
| 2-4 times/week |  | 21% (395) | 21% (387) | 18% (173) |
| 5+ times/week |  | 35% (653) | 39% (736) | 8% (75) |
| **Team/Partner Sport Participation** | **%** | 38% (753) | 57% (1157) | 22% (209) |
| **Hypertension or Antihypertensive Use** | **%** | 14% (286) | 26% (527) | 85% (810) |
| **History of Cardiovascular disease (Excl. Stroke)** | **%** | <1% (3) | <1% (9) | 22% (212) |
| **History of Stroke** | **%** | <1% (5) | <1% (9) | 9% (85) |
| **Limiting Disability** |  | 14% (287) | 9% (195) | 29% (287) |
| **Diabetes** |  | 3% (55) | 4% (79) | 20% (191) |
| **Depression or Anxiety** | **%** | 17% (343) | 13% (266) | 19% (180) |
| **Social Participation (Meeting Family)** | **%** |  |  | - |
| Never or less than yearly |  | 1% (28) | 2% (45) | - |
| Once or twice/year |  | 4% (77) | 6% (124) | - |
| Every few months |  | 12% (247) | 17% (353) | - |
| Once or twice/month |  | 22% (438) | 25% (509) | - |
| Once or twice/week |  | 35% (703) | 33% (689) | - |
| Three or more times a week |  | 26% (513) | 15% (315) | - |
| **Social Participation (Meeting Friends)** | % |  |  |  |
| Never |  | 1% (28) | 2% (35) |  |
| Less than yearly |  | 1% (15) | 2% (32) | - |
| Once or twice/year |  | 21% (43) | 3% (55) | - |
| Every few months |  | 9% (188) | 14% (275) | - |
| Once or twice/month |  | 33% (653) | 31% (627) | - |
| Once or twice/week |  | 39% (785) | 38% (765) | - |
| Three or more times a week |  | 15% (294) | 14% (275) | - |
| **Social Participation (Hours Meeting family, friends and participating in other leisure activities)** | **Median (IQR)** | - | - | 10 (4-18) |

**Supplementary Table S3. Cognitive test scores in BCS70 participants**

| **Cognition Test** | **BCS70 (Female)** |  | **BCS70 (Male)** | **Comparison** |
| --- | --- | --- | --- | --- |
|  | ***N=2006*** |  | ***N=2,035*** |  |
|  | Median (IQR) |  | Median (IQR) | *p-value* |
| Processing Speed | 353 (296-407) |  | 329 (278-393) | <0.001 |
| Processing Accuracy | 3 (2-6) |  | 3 (1-5) | 0.858 |
| Verbal Fluency | 24 (20-28) |  | 24 (20-28) | 0.394 |
| Recall (immediate) | 7 (6-8) |  | 7 (6-8) | 0.011 |
| Recall (delayed) | 6 (5-7) |  | 6 (4-7) | <0.001 |

Comparison by t-test for normally distributed measures and Mann-Whitney U-test

test for non-normal distributions (processing accuracy).

**Supplementary Table S4. TYM test scores in BRHS participants and stratified by cognition category based on TYM cut points.**

| **BRHS Participant TYM score and stratified by cognition category.** | | | |  |
| --- | --- | --- | --- | --- |
|  |  | **Total Sample** | **Mild/Severe Cognitive Impairment** | **Normal Cognition** |
|  |  | ***N=963*** | ***N=422*** | ***N=535*** |
| **TYM Score** | **Median, IQR** | 41 (37-43) | 36 (32-39) | 43 (42-44) |

|  | ***Model 1: Unadjusted*** | | | | ***Model 2: Adjustment for Socioeconomic class, region and education.*** | | | | ***Model 3: Additionally adjusts for disability status, BMI, history of stroke, CVD, hypertension, diabetes, smoker status, alcohol consumption.*** | | | | ***Model 4: additionally adjusts for physical activity, wear time and wear season.*** | | | | ***Model 5: additionally adjusts for other social engagement.*** | | | |
| --- | --- | --- | --- | --- | --- | --- | --- | --- | --- | --- | --- | --- | --- | --- | --- | --- | --- | --- | --- | --- |
|  | ***Coef.*** | ***95% CI*** | | ***p-value*** | ***Coef.*** | ***95% CI*** | | ***p-value*** | ***Coef.*** | ***95% CI*** | | ***p-value*** | ***Coef.*** | ***95% CI*** | | ***p-value*** | ***Coef.*** | ***95% CI*** | | ***p-value*** |
| ***No sport participation (Ref)*** | **-** | **-** | **-** | **-** | **-** | **-** | **-** | **-** | **-** | **-** | **-** | **-** | **-** | **-** | **-** | **-** | **-** | **-** | **-** | **-** |
| **Less than once a month** | 0.158 | -0.043 | 0.359 | 0.124 | 0.056 | -0.164 | 0.277 | 0.616 | 0.032 | -0.189 | 0.253 | 0.774 | 0.031 | -0.190 | 0.252 | 0.782 | 0.028 | -0.195 | 0.251 | 0.807 |
| **1-4 times/ month** | 0.191 | 0.042 | 0.340 | **0.012** | 0.174 | 0.007 | 0.341 | **0.042** | 0.138 | -0.030 | 0.306 | 0.107 | 0.139 | -0.029 | 0.307 | 0.106 | 0.141 | -0.029 | 0.311 | 0.104 |
| **2-4 times/ week** | 0.392 | 0.246 | 0.537 | **<0.001** | 0.202 | 0.033 | 0.372 | **0.019** | 0.162 | -0.010 | 0.333 | 0.065 | 0.168 | -0.005 | 0.340 | 0.057 | 0.165 | -0.009 | 0.340 | 0.063 |
| **5+ times/week** | 0.364 | 0.233 | 0.496 | **<0.001** | 0.228 | 0.078 | 0.378 | **0.003** | 0.177 | 0.023 | 0.332 | **0.025** | 0.184 | 0.029 | 0.340 | **0.020** | 0.172 | 0.014 | 0.331 | **0.033** |

**Point Estimates for Associated figures.**

**Supplementary Table S5. Linear Regression of 1970 British Cohort Study (female) participant sports participation frequency and composite cognition z-scores.**

**Supplementary Table S6. Linear Regression of 1970 British Cohort Study (male) participant sports participation frequency and cognition z-scores.**

|  | ***Model 1: Unadjusted*** | | | | ***Model 2: Adjustment for Socioeconomic class, region and education.*** | | | | ***Model 3: Additionally adjusts for disability status, BMI, history of stroke, CVD, hypertension, diabetes, smoker status, alcohol consumption.*** | | | | ***Model 4: additionally adjusts for physical activity, wear time and wear season.*** | | | | ***Model 5: additionally adjusts for other social engagement.*** | | | |
| --- | --- | --- | --- | --- | --- | --- | --- | --- | --- | --- | --- | --- | --- | --- | --- | --- | --- | --- | --- | --- |
|  | ***Coef.*** | ***95% CI*** | | ***p-value*** | ***Coef.*** | ***95% CI*** | | ***p-value*** | ***Coef.*** | ***95% CI*** | | ***p-value*** | ***Coef.*** | ***95% CI*** | | ***p-value*** | ***Coef.*** | ***95% CI*** | | ***p-value*** |
| ***No sport participation (Ref)*** | **-** | **-** | **-** | **-** | **-** | **-** | **-** | **-** | **-** | **-** | **-** | **-** | **-** | **-** | **-** | **-** | **-** | **-** | **-** | **-** |
| **Less than once a month** | 0.105 | -0.111 | 0.320 | 0.342 | -0.032 | -0.274 | 0.211 | 0.798 | -0.075 | -0.318 | 0.168 | 0.547 | -0.082 | -0.326 | 0.161 | 0.506 | -0.099 | -0.342 | 0.145 | 0.426 |
| **1-4 times/ month** | 0.243 | 0.084 | 0.402 | **0.003** | 0.160 | -0.015 | 0.336 | 0.074 | 0.109 | -0.068 | 0.286 | 0.226 | 0.117 | -0.060 | 0.294 | 0.194 | 0.083 | -0.095 | 0.261 | 0.359 |
| **2-4 times/ week** | 0.455 | 0.297 | 0.614 | **<0.001** | 0.242 | 0.059 | 0.425 | **0.009** | 0.182 | -0.003 | 0.367 | 0.054 | 0.187 | 0.002 | 0.372 | **0.047** | 0.163 | -0.023 | 0.349 | 0.085 |
| **5+ times/week** | 0.483 | 0.340 | 0.626 | **<0.001** | 0.274 | 0.112 | 0.437 | **0.001** | 0.194 | 0.027 | 0.362 | **0.023** | 0.205 | 0.036 | 0.373 | **0.017** | 0.183 | 0.013 | 0.353 | **0.035** |

**Supplementary Table S7. Linear Regression of British Regional Heart Study (male) participant sports participation frequency and TYM z-scores.**

|  | ***Model 1: Age-adjusted*** | | | | ***Model 2: Adjustment for Social class, region and education.*** | | | | ***Model 3: Additionally adjusts for disability status, BMI, history of stroke, CVD, hypertension, diabetes, smoker status, alcohol consumption.*** | | | | ***Model 4: additionally adjusts for physical activity, wear time and wear season.*** | | | | ***Model 5: additionally adjusts for other social engagement.*** | | | |
| --- | --- | --- | --- | --- | --- | --- | --- | --- | --- | --- | --- | --- | --- | --- | --- | --- | --- | --- | --- | --- |
|  | ***Coef.*** | ***95% CI*** | | ***p-value*** | ***Coef.*** | ***95% CI*** | | ***p-value*** | ***Coef.*** | ***95% CI*** | | ***p-value*** | ***Coef.*** | ***95% CI*** | | ***p-value*** | ***Coef.*** | ***95% CI*** | | ***p-value*** |
| ***No sport participation (Ref)*** | **-** | **-** | **-** | **-** | **-** | **-** | **-** | **-** | **-** | **-** | **-** | **-** | **-** | **-** | **-** | **-** | **-** | **-** | **-** | **-** |
| **Less than once a month** | 0.165 | -0.127 | 0.457 | 0.267 | 0.085 | -0.193 | 0.362 | 0.550 | 0.067 | -0.216 | 0.350 | 0.643 | 0.075 | -0.207 | 0.357 | 0.603 | 0.073 | -0.210 | 0.355 | 0.615 |
| **1-4 times/ month** | 0.366 | 0.137 | 0.595 | **0.002** | 0.266 | 0.048 | 0.485 | **0.017** | 0.264 | 0.041 | 0.487 | **0.020** | 0.259 | 0.037 | 0.482 | **0.022** | 0.258 | 0.035 | 0.481 | **0.023** |
| **2-4 times/ week** | 0.261 | 0.091 | 0.430 | **0.003** | 0.142 | -0.021 | 0.304 | 0.087 | 0.129 | -0.038 | 0.296 | 0.131 | 0.111 | -0.057 | 0.280 | 0.195 | 0.107 | -0.062 | 0.277 | 0.214 |
| **5+ times/week** | 0.205 | -0.035 | 0.444 | 0.094 | 0.157 | -0.070 | 0.385 | 0.175 | 0.169 | -0.066 | 0.403 | 0.158 | 0.124 | -0.115 | 0.363 | 0.308 | 0.119 | -0.121 | 0.360 | 0.330 |

| **Supplementary Table S8. Linear Regression of 1970 British Cohort Study (female) participant sports participation frequency and composite cognition z-scores.** | | | | | | | | | | | | | | | | | | | | |
| --- | --- | --- | --- | --- | --- | --- | --- | --- | --- | --- | --- | --- | --- | --- | --- | --- | --- | --- | --- | --- |
|  |  |  |  |  |  |  |  |  |  |  |  |  |  |  |  |  |  |  |  |  |
|  | ***Model 1: Unadjusted*** | | | | ***Model 2: Adjustment for Socioeconomic class, region and education.*** | | | | ***Model 3: Additionally adjusts for disability status, BMI, history of stroke, CVD, hypertension, diabetes, smoker status, alcohol consumption.*** | | | | ***Model 4: additionally adjusts for physical activity, wear time and wear season.*** | | | | ***Model 5: additionally adjusts for other social engagement.*** | | | |
|  | ***Coef.*** | ***95% CI*** | | ***p-value*** | ***Coef.*** | ***95% CI*** | | ***p-value*** | ***Coef.*** | ***95% CI*** | | ***p-value*** | ***Coef.*** | ***95% CI*** | | ***p-value*** | ***Coef.*** | ***95% CI*** | | ***p-value*** |
| ***No sport participation (Ref)*** | **-** | **-** | **-** | **-** | **-** | **-** | **-** | **-** | **-** | **-** | **-** | **-** | **-** | **-** | **-** | **-** | **-** | **-** | **-** | **-** |
| **Other Sports** | 0.165 | 0.049 | 0.281 | **0.005** | 0.068 | -0.062 | 0.197 | 0.304 | 0.039 | -0.092 | 0.169 | 0.562 | 0.042 | -0.089 | 0.172 | 0.533 | 0.037 | -0.095 | 0.169 | 0.583 |
| **Team/Partner Sports** | 0.326 | 0.208 | 0.444 | **<0.001** | 0.250 | 0.112 | 0.387 | **<0.001** | 0.207 | 0.067 | 0.347 | **0.004** | 0.208 | 0.068 | 0.348 | **0.004** | 0.207 | 0.064 | 0.349 | **0.004** |

| **Supplementary Table S9. Linear Regression of 1970 British Cohort Study (male) participant sports participation frequency and composite cognition z-scores.** | | | | | | | | | | | | | | | | | | | | |
| --- | --- | --- | --- | --- | --- | --- | --- | --- | --- | --- | --- | --- | --- | --- | --- | --- | --- | --- | --- | --- |
|  |  |  |  |  |  |  |  |  |  |  |  |  |  |  |  |  |  |  |  |  |
|  | ***Model 1: Unadjusted*** | | | | ***Model 2: Adjustment for Socioeconomic class, region and education.*** | | | | ***Model 3: Additionally adjusts for disability status, BMI, history of stroke, CVD, hypertension, diabetes, smoker status, alcohol consumption.*** | | | | ***Model 4: additionally adjusts for physical activity, wear time and wear season.*** | | | | ***Model 5: additionally adjusts for other social engagement.*** | | | |
|  | ***Coef.*** | ***95% CI*** | | ***p-value*** | ***Coef.*** | ***95% CI*** | | ***p-value*** | ***Coef.*** | ***95% CI*** | | ***p-value*** | ***Coef.*** | ***95% CI*** | | ***p-value*** | ***Coef.*** | ***95% CI*** | | ***p-value*** |
| ***No sport participation (Ref)*** | **-** | **-** | **-** | **-** | **-** | **-** | **-** | **-** | **-** | **-** | **-** | **-** | **-** | **-** | **-** | **-** | **-** | **-** | **-** | **-** |
| **Other Sports** | 0.309 | 0.171 | 0.446 | **<0.001** | 0.145 | -0.009 | 0.299 | 0.065 | 0.091 | -0.064 | 0.246 | 0.250 | 0.096 | -0.059 | 0.251 | 0.224 | 0.075 | -0.081 | 0.230 | 0.348 |
| **Team/Partner Sports** | 0.431 | 0.306 | 0.555 | **<0.001** | 0.286 | 0.147 | 0.426 | **<0.001** | 0.219 | 0.077 | 0.361 | **0.003** | 0.225 | 0.083 | 0.367 | **0.002** | 0.205 | 0.061 | 0.349 | **0.005** |

| **Supplementary Table S10. Linear Regression of British Regional Heart Study participant sports participation frequency and TYM z-scores.** | | | | | | | | | | | | | | | | | | | | |  |
| --- | --- | --- | --- | --- | --- | --- | --- | --- | --- | --- | --- | --- | --- | --- | --- | --- | --- | --- | --- | --- | --- |
|  |  |  |  |  |  |  |  |  |  |  |  |  |  |  |  |  |  |  |  |  |  |
|  | ***Model 1: Age-adjusted*** | | | | ***Model 2: Adjustment for Social class, region and education.*** | | | | ***Model 3: Additionally adjusts for disability status, BMI, history of stroke, CVD, hypertension, diabetes, smoker status, alcohol consumption.*** | | | | ***Model 4: additionally adjusts for physical activity, wear time and wear season.*** | | | | ***Model 5: additionally adjusts for other social engagement.*** | | | |  |
|  | ***Coef.*** | ***95% CI*** | | ***p-value*** | ***Coef.*** | ***95% CI*** | | ***p-value*** | ***Coef.*** | ***95% CI*** | | ***p-value*** | ***Coef.*** | ***95% CI*** | | ***p-value*** | ***Coef.*** | ***95% CI*** | | ***p-value*** |  |
| ***No sport participation (Ref)*** | **-** | **-** | **-** | **-** | **-** | **-** | **-** | **-** | **-** | **-** | **-** | **-** | **-** | **-** | **-** | **-** | **-** | **-** | **-** | **-** |  |
| **Other Sports** | 0.305 | 0.142 | 0.469 | **<0.001** | 0.154 | -0.003 | 0.312 | 0.055 | 0.146 | -0.017 | 0.308 | 0.079 | 0.119 | -0.045 | 0.283 | 0.154 | 0.117 | -0.048 | 0.282 | 0.165 |  |
| **Team/Partner Sports** | 0.237 | 0.079 | 0.395 | **0.003** | 0.182 | 0.031 | 0.334 | **0.019** | 0.172 | 0.016 | 0.328 | **0.031** | 0.163 | 0.006 | 0.321 | **0.042** | 0.161 | 0.002 | 0.319 | **0.047** |  |

**Supplementary Table S11. Binary Logistic Regression of British Regional Heart Study participant (male) sports participation frequency and categorised TYM scores (Ref: Normal Cognition)**

|  | ***Model 1: Age-Adjusted*** | | | | ***Model 2: Adjustment for Social class, region and education.*** | | | | ***Model 3: Additionally adjusts for disability status, BMI, history of stroke, CVD, hypertension, diabetes, smoker status, alcohol consumption.*** | | | | ***Model 4: additionally adjusts for physical activity, wear time and wear season.*** | | | | ***Model 5: additionally adjusts for other social engagement.*** | | | |
| --- | --- | --- | --- | --- | --- | --- | --- | --- | --- | --- | --- | --- | --- | --- | --- | --- | --- | --- | --- | --- |
|  | ***Coef.*** | ***95% CI*** | | ***p-value*** | ***Coef.*** | ***95% CI*** | | ***p-value*** | ***Coef.*** | ***95% CI*** | | ***p-value*** | ***Coef.*** | ***95% CI*** | | ***p-value*** | ***Coef.*** | ***95% CI*** | | ***p-value*** |
| ***No sport participation (Ref)*** | **-** | **-** | **-** | **-** | **-** | **-** | **-** | **-** | **-** | **-** | **-** | **-** | **-** | **-** | **-** | **-** | **-** | **-** | **-** | **-** |
| **Less than once a month** | 0.080 | -0.065 | 0.226 | 0.280 | 0.056 | -0.087 | 0.199 | 0.443 | 0.065 | -0.081 | 0.210 | 0.382 | 0.068 | -0.077 | 0.213 | 0.360 | 0.065 | -0.081 | 0.210 | 0.383 |
| **1-4 times/ month** | 0.169 | 0.054 | 0.283 | **0.004** | 0.141 | 0.029 | 0.254 | **0.014** | 0.149 | 0.035 | 0.264 | **0.011** | 0.147 | 0.033 | 0.261 | **0.012** | 0.145 | 0.031 | 0.260 | **0.013** |
| **2-4 times/ week** | 0.168 | 0.083 | 0.252 | **<0.001** | 0.126 | 0.042 | 0.210 | **0.003** | 0.125 | 0.039 | 0.211 | **0.004** | 0.117 | 0.031 | 0.204 | **0.008** | 0.112 | 0.025 | 0.199 | **0.012** |
| **5+ times/week** | 0.110 | -0.009 | 0.229 | 0.070 | 0.096 | -0.021 | 0.213 | 0.108 | 0.100 | -0.020 | 0.220 | 0.104 | 0.081 | -0.042 | 0.204 | 0.197 | 0.074 | -0.049 | 0.198 | 0.238 |

| **Supplementary Table S12. Binary Logistic Regression of British Regional Heart Study participant (male) sports participation frequency and categorised TYM scores (Ref: Normal Cognition)** | | | | | | | | | | | | | | | | | | | | |  |
| --- | --- | --- | --- | --- | --- | --- | --- | --- | --- | --- | --- | --- | --- | --- | --- | --- | --- | --- | --- | --- | --- |
|  |  |  |  |  |  |  |  |  |  |  |  |  |  |  |  |  |  |  |  |  |  |
|  | ***Model 1: Age-adjusted*** | | | | ***Model 2: Adjustment for Social class, region and education.*** | | | | ***Model 3: Additionally adjusts for disability status, BMI, history of stroke, CVD, hypertension, diabetes, smoker status, alcohol consumption.*** | | | | ***Model 4: additionally adjusts for physical activity, wear time and wear season.*** | | | | ***Model 5: additionally adjusts for other social engagement.*** | | | |  |
|  | ***Coef.*** | ***95% CI*** | | ***p-value*** | ***Coef.*** | ***95% CI*** | | ***p-value*** | ***Coef.*** | ***95% CI*** | | ***p-value*** | ***Coef.*** | ***95% CI*** | | ***p-value*** | ***Coef.*** | ***95% CI*** | | ***p-value*** |  |
| ***No sport participation (Ref)*** | **-** | **-** | **-** | **-** | **-** | **-** | **-** | **-** | **-** | **-** | **-** | **-** | **-** | **-** | **-** | **-** | **-** | **-** | **-** | **-** |  |
| **Other Sports** | 0.149 | 0.068 | 0.231 | **<0.001** | 0.097 | 0.016 | 0.178 | **0.019** | 0.098 | 0.015 | 0.181 | **0.021** | 0.087 | 0.003 | 0.172 | **0.043** | 0.083 | -0.002 | 0.168 | 0.055 |  |
| **Team/Partner Sports** | 0.137 | 0.058 | 0.216 | **0.001** | 0.120 | 0.042 | 0.198 | **0.003** | 0.123 | 0.043 | 0.203 | **0.003** | 0.118 | 0.037 | 0.199 | **0.004** | 0.114 | 0.033 | 0.196 | **0.006** |  |

**Supplementary Figure S3. Association of sporting LTPA participation and sporting LTPA
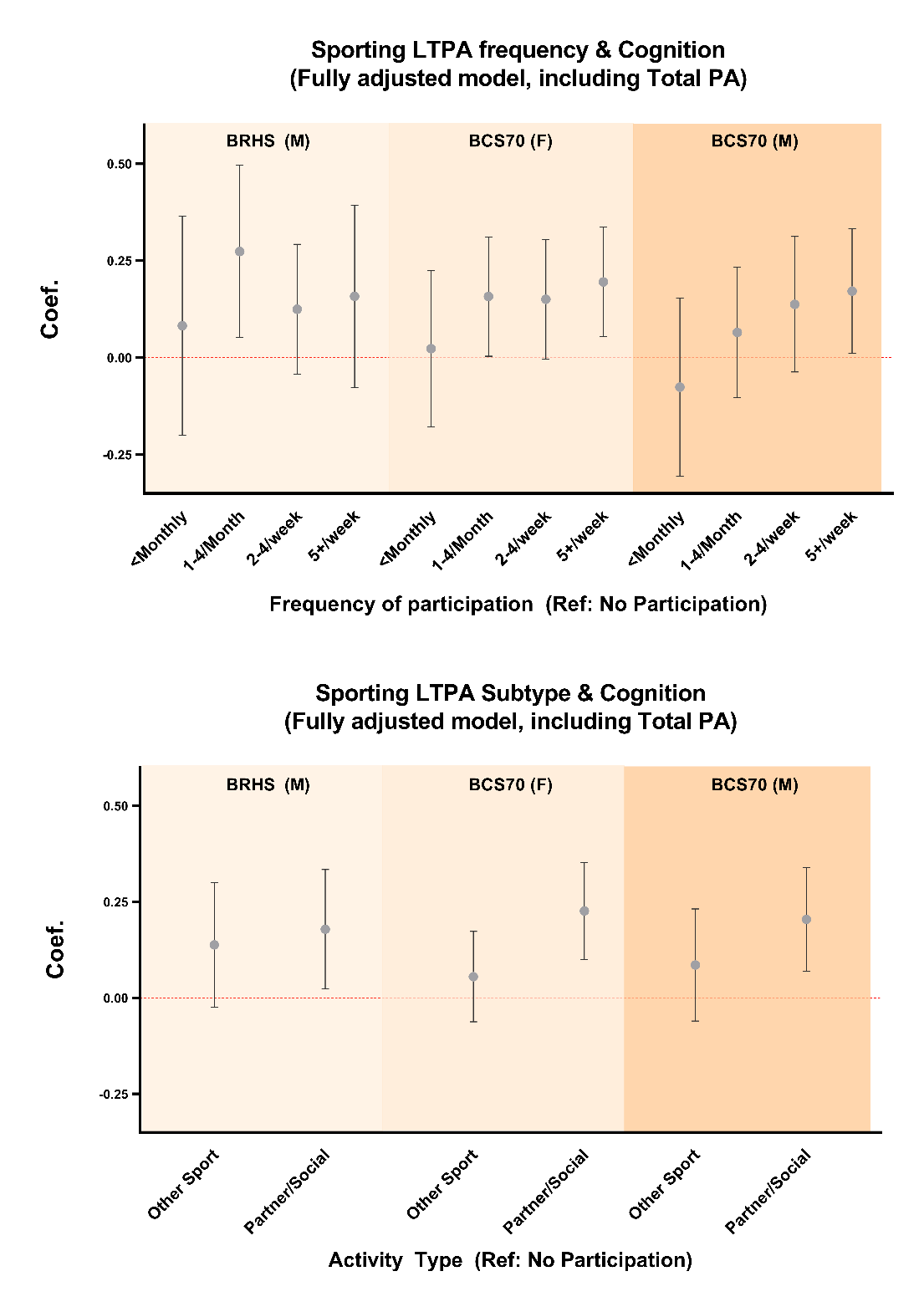
 participation context with cognition (Fully adjusted models replacing MVPA with Total PA).**

| **Supplementary Table S13. Linear Regression of British Regional Heart Study participant (male) sports participation frequency and TYM z-scores** | | | | |
| --- | --- | --- | --- | --- |
|  | **Coef.** | **95% CI** | | **p-value** |
| None (REF) | - | - | - | - |
| <Monthly | 0.08 | -0.20 | 0.37 | 0.57 |
| 1-4/Month | **0.27** | 0.05 | 0.50 | **0.02** |
| 2-4/week | 0.12 | -0.04 | 0.29 | 0.14 |
| 5+/week | 0.16 | -0.08 | 0.39 | 0.19 |

| **Supplementary Table S14. Linear Regression of British Regional Heart Study participant (male) sports participation type and TYM z-scores.** | | | | |
| --- | --- | --- | --- | --- |
|  | **Coef.** | **95% CI** |  | **p-value** |
| None (REF) | - | - | - | - |
| Other Sport | 0.14 | -0.02 | 0.30 | 0.10 |
| Partner/Social | 0.18 | 0.02 | 0.33 | **0.02** |

| **Supplementary Table S15. Linear Regression of 1970 British Cohort Study participant (female) sports participation frequency and cognition z-scores** | | | | |
| --- | --- | --- | --- | --- |
|  | **Coef.** | **95% CI** | | **p-value** |
| None (REF) | - | - | - | - |
| <Monthly | 0.02 | -0.18 | 0.22 | 0.82 |
| 1-4/Month | **0.16** | 0.00 | 0.31 | **0.04** |
| 2-4/week | 0.15 | 0.00 | 0.30 | 0.06 |
| 5+/week | **0.20** | 0.05 | 0.34 | **0.01** |

| **Supplementary Table S16. Linear Regression of 1970 British Cohort Study participant (female) sports participation type and cognition z-scores** | | | | |
| --- | --- | --- | --- | --- |
|  | **Coef.** | **95% CI** |  | **p-value** |
| None (REF) | - | - | - | - |
| Other Sport | 0.06 | -0.06 | 0.17 | 0.36 |
| Partner/Social | 0.23 | 0.10 | 0.35 | **<0.01** |

| **Supplementary Table S17. Linear Regression of 1970 British Cohort Study participant (male) sports participation frequency and cognition z-scores** | | | | |
| --- | --- | --- | --- | --- |
|  | **Coef.** | **95% CI** | | **p-value** |
| None (REF) | - | - | - | - |
| <Monthly | -0.08 | -0.30 | 0.15 | 0.52 |
| 1-4/Month | **0.07** | -0.10 | 0.23 | **0.45** |
| 2-4/week | 0.14 | -0.04 | 0.31 | 0.12 |
| 5+/week | **0.17** | 0.01 | 0.33 | **0.04** |

| **Supplementary Table S18. Linear Regression of 1970 British Cohort Study participant (male) sports participation type and cognition z-scores** | | | | |
| --- | --- | --- | --- | --- |
|  | **Coef.** | **95% CI** |  | **p-value** |
| None (REF) | - | - | - | - |
| Other Sport | 0.09 | -0.06 | 0.23 | 0.25 |
| Partner/Social | 0.20 | 0.07 | 0.34 | **<0.01** |
